# The role of living donor liver transplantation in treating intrahepatic cholangiocarcinoma

**DOI:** 10.3389/fonc.2024.1404683

**Published:** 2024-05-21

**Authors:** Wellington Andraus, Gabriela Ochoa, Rodrigo Bronze de Martino, Rafael Soares Nunes Pinheiro, Vinicius Rocha Santos, Liliana Ducatti Lopes, Rubens Macedo Arantes Júnior, Daniel Reis Waisberg, Alexandre Chagas Santana, Francisco Tustumi, Luiz Augusto Carneiro D’Albuquerque

**Affiliations:** Department of Gastroenterology, Transplantation Unit, Universidade de São Paulo, São Paulo, Brazil

**Keywords:** cholangiocarcinoma, liver transplantation, living donors, hepatectomy, liver neoplasms

## Abstract

**Introduction:**

Intrahepatic cholangiocarcinoma (iCC) is the liver’s second most common neoplasm. Until now, surgery is the only curative option, but only 35% of the cases are considered resectable at the diagnosis, with a post-resection survival of around 30%. Advancements in surgical techniques and perioperative care related to liver transplantation (LT) have facilitated the expansion of indications for hepatic neoplasms.

**Method:**

This study is a comprehensive review of the global experience in living donor LT (LDLT) for treating iCC and describes our first case of LDLT for an unresectable iCC.

**Results:**

While exploring LT for intrahepatic cholangiocarcinoma dates to the 1990s, the initial outcomes were discouraging, marked by poor survival and high recurrence rates. Nevertheless, contemporary perspectives underscore a reinvigorated emphasis on extending the frontiers of LT indications within the context of the “oncologic era.” The insights gleaned from examining explants, wherein incidental iCC was categorized as hepatocellular carcinoma in the preoperative period, have demonstrated comparable survival rates to small hepatocellular carcinoma. These findings substantiate the potential viability of LT as a curative alternative for iCC. Another investigated scenario pertains to “unresectable tumors with favorable biological behavior,” LT presents a theoretical advantage by providing free margins without the concern of a small future liver remnant. The constraint of organ shortage persists, particularly in nations with low donation rates. LDLT emerges as a viable and secure alternative for treating iCC.

**Conclusion:**

LDLT is an excellent option for augmenting the graft pool, particularly in carefully selected patients.

## Introduction

Cancer is considered a contraindication to transplant for most organs. However, liver transplantation can be a curative strategy for some malignancies. The expansion of indications in the new era of oncologic transplantation was made possible due to the improvement of the perioperative outcomes and postoperative treatment and the long experience in treating hepatocellular carcinoma (HCC) (1).

HCC is an accepted indication for transplantation. However, tumor size and standardized multidisciplinary treatment protocols are necessary to ensure optimal patient outcomes. On the other hand, cholangiocarcinoma (CC) is still a controversial indication worldwide (2).

CC is a highly lethal epithelial cell malignancy along the biliary tree and within the hepatic parenchyma. The CCAs are divided into three subtypes depending on their anatomical location: intrahepatic (iCC), hilar (hCC), and distal (dCC). CC is the second most common primary hepatic malignancy, after HCC, comprising approximately 15% of all primary liver tumors and 3% of gastrointestinal cancers. Despite its rarity, the incidence (0.3–6 per 100,000 inhabitants per year) and mortality (1–6 per 100,000 inhabitants per year globally) of CC have witnessed a discernible surge worldwide over recent decades, constituting a global health challenge (3). The prognosis for this malignancy remains bleak, with a 5-year survival rate ranging from 7 to 20% and a notable risk for tumor recurrence after resection. Surgery is the treatment of choice for early-stage tumors, regardless of the anatomical type. However, only 35% of the patients are eligible for surgical treatment, and there is a high rate of postoperative recurrence (4).

The iCC subgroup represents 10–20% of all CC and arises above the second-order bile ducts. The prognosis is usually dismal, with a reported 5-year overall survival of 10% to 35%. However, the prognosis is strictly related to stage and molecular profiles (5). The recommended treatment for advanced stages is chemotherapy combined with immunotherapy, with a median overall survival of less than one year (6, 7).

Liver transplantation (LT) offers a theoretical advantage to allowing surgical radicality. LT avoids the risk of the liver’s future small remanent and cures the underlying liver disease in cases of cirrhosis. The initial international experience with LT for iCC in the 1990s, particularly in advanced cases, yielded suboptimal outcomes marked by compromised survival rates and heightened morbidity (8). Currently, emphasis has shifted towards meticulous candidate selection, considering factors such as tumor size and biological behavior to identify individuals who are more likely to benefit from LT (9–11).

Due to organ shortage and increasing organ demand in most countries worldwide, the allocation of liver grafts is always meticulously analyzed and discussed before any expansion of transplant indications in cases of deceased donor LT. In 2022, Brazil achieved a donation rate of 16.5 per million population (pmp), surpassing certain neighboring countries but still trailing behind nations with more robust organ donation rates, such as Spain and the USA, which boast 46 and 44 pmp per year, respectively (12). In this context, living donor liver transplantation (LDLT) is an excellent option in regions where the allocation system does not allow a real opportunity to get an organ on the waiting list. In countries with low donation rates, such as Latin America, the LDLT represents a good solution for oncological indications in LT.

While LT has become one of the main treatment alternatives for hCC, LT is still not universally accepted for iCC (13). We presented a comprehensive review of the international experience with LTLT for iCC and reported our first case of LTLT for iCC (14, 15).

## Initial experience

In the ‘90s and the beginning of 2000, during the expansion of the indications of LT, a few cases of unresectable liver malignancies were treated with LT. However, the oncologic results were poor, with a high recurrence rate and a low overall survival. Goldstein et al. reported 17 patients with cholangiocarcinoma submitted to LT in 1993. Three of them were excluded due to premature mortality. Among the remaining 14 patients, 11 experienced recurrences during the follow-up, and within one year, seven succumbed to disease progression. The 1-year survival rate within this series was 53%, with a corresponding disease-free survival rate of 40% (16). In 2000, Meyer et al. reported 207 cases of LT for unresectable CC or cholangiohepatoma. The survival of 1, 2, and 5-year were 72, 48, and 23%. Fifty-one percent of patients presented a recurrence of their tumors after transplantation, and 84% of recurrences occurred within two years of transplantation (17).

## LT in incidental lesions of iCC in the explant or HCC misdiagnosis

Regarding HCC, the LT is the best treatment to cure cases under biological and size selection criteria, with an excellent overall survival at five years, reaching 80%. Consequently, in the contemporary landscape, HCC is one of the main indications for LT worldwide, serving as a cornerstone in oncologic transplantation support. Since it is unnecessary to perform a biopsy for suspected tumors, a small percentage of misdiagnosed lesions had been included for transplant over time. Sapisochin et al. published in 2011 the analysis of 14 explant specimens from 302 patients (4.6%) who underwent LT intentionally for HCC that showed mixed HCC-CC or iCC, with 10 falling into the latter category. After a median follow-up period of 32 months, 8 of the 14 patients (57%) suffered from tumor recurrence, and the median disease-free survival time was eight months (18). In 2014, the same author advocated incorporating a size criterion, following a Spanish-matched cohort multicenter study comparing 27 iCC with 54 HCC. Patients with uninodular tumors of 2 cm or smaller in the study group had similar 1-, 3-, and 5-year survival rates with the HCC control group (92%, 83%, 62% *vs.* 100%, 80%, 80%; P = 0.4). In contrast, patients with multinodular or uninodular tumors larger than 2 cm had worse 1-, 3-, and 5-year survival rates than their controls (80%, 66%, and 61% *vs.* 99%, 96%, and 90%; P < 0.001) (19).

In 2016, the iCC International Consortium introduced the term “very early iCC,” denoting single tumors with a size of 2 cm or smaller. Their findings revealed compelling survival rates for this category, with percentages of 93%, 84%, and 65% at 1, 3, and 5 years, respectively. In contrast, the advanced iCC group, characterized by a single tumor larger than 2 cm or multifocal disease, exhibited survival rates of 79%, 50%, and 45% at the respective time intervals (20).

The discovery of incidental iCC lesions in LT explants and their subsequent analysis has rekindled interest in using LT as a viable treatment modality for this disease.

## LT for unresectable advanced iCC

Currently, unresectable intrahepatic tumors are usually treated with systemic chemotherapy. The regimen with gemcitabine and cisplatin yields an overall survival rate of only 18.9 months and a progression-free survival duration of 11.1 months (21).

In recent years, significant advances have been achieved in understanding iCC. The new distinction between small and large duct tumors, coupled with identifying mutations and associated risk factors for each subtype, seems to be the key to advancement in treatment. This nuanced distinction holds promise for refining the selection of cases based on their biological behavior and, eventually, enabling the identification of candidates for LT in the context of unresectable tumors, with the ultimate goal of achieving curative outcomes (22–24).

The experience with pCC showed that neoadjuvant therapy followed by LT results in a long-term survival advantage in patients without disease progression (13). For iCC the first case series of neoadjuvant treatment was reported by Hong at all in 2011. In their published experience, encompassing 25 transplanted patients with iCC, they detailed that nine of these individuals underwent neoadjuvant and adjuvant therapy. When comparing patients who received combined neoadjuvant and adjuvant therapy to those who received no therapy or only adjuvant treatment, a discernible advantage in terms of survival emerged for the group that underwent both treatments (47% *vs.* 20% *vs.* 33%, respectively; P = 0.03) (25).

Lunsford et al. reported that 12 patients underwent evaluation for potential LT and were diagnosed with unresectable iCC in 2018. These individuals underwent an extensive neoadjuvant protocol involving gemcitabine-based chemotherapy and, in some cases, a subsequent second or third-line regimen. At the time of publication, six of the patients had undergone transplantation. The survival rates at 1, 3, and 5 years were 100%, 83.3%, and 83.3%, respectively. However, during the follow-up period, disease recurrence occurred in three patients, constituting a recurrence rate of 50% (26).

Certainly, neoadjuvant therapies play a crucial role in reducing the likelihood of recurrence and serve as a valuable assessment tool for evaluating the favorable biological behavior of tumors. Moreover, they contribute to the selective identification of patients who stand to benefit from liver transplantation as a viable treatment strategy for tumors.

## LDLT for iCC

The limited availability of donor organs in comparison to the growing demand has intensified concerns about the allocation of grafts, mainly when used for innovative indications, especially in the context of cancer. This concern is further exacerbated by the potential for disease recurrence and associated mortality, prompting careful consideration of resource allocation in these circumstances.

In most countries, the shortage of organs due to a low rate of donations and the dropout in the waiting list of patients with current indications does not allow the expansion of LT to treat new conditions. Like most nations, Brazil has adopted the Model for End-Stage Liver Disease (MELD) score allocation system. This system grants exception points for specific criteria in HCC cases. However, it does not permit the inclusion of patients with other primary malignant liver neoplasms, thereby limiting the chances for individuals with iCC to be added to the waiting list (27).

In this context, LDLT stands as a safety-assured strategy for the global expansion of liver transplantation. LDLT has become an effective treatment option to overcome the deceased donor organ shortage and an excellent alternative to treat selected oncologic cases (28).

The LDLT for HCC has been widely used, especially in oriental countries. For instance, Kyushu University reported 90 cases of LT in 2017, employing expanded criteria based on size and des-γ-carboxy prothrombin levels in HCC, utilizing living donor grafts. This cohort’s 5-year overall patient survival rate was an impressive 89%. Similarly, the pilot study conducted by the Barcelona Clinic Liver Group 2018 utilized extended criteria, incorporating factors such as size, number, and downstaging as selection criteria. The study, comprising 22 patients with a follow-up duration of 81 months, reported survival rates of 95.5%, 86.4%, 80.2%, and 66.8% at 1, 3, 5, and 10 years, respectively. These findings underscore the success of LDLT in managing HCC and the potential applicability of expanded criteria in diverse clinical settings (29, 30).

The LDLT strategy offers a valuable solution for iCC, avoiding the long waiting list. This strategy proves beneficial not only in early iCC accompanied by underlying cirrhosis but also in advanced stages exhibiting a favorable response post-neoadjuvant therapy. The expeditious nature of this approach is crucial, considering the observed higher dropout rates in iCC patients.

While the literature on this topic remains limited, our review revealed eight articles documenting cases of LDLT for iCC. Among them, five specifically addressed living donors as a primary focus, while three presented a more comprehensive series encompassing cases of LDLT. Notably, only two reports featured more than two cases. The most extensive series was published by Sierra et al., focusing on LT outcomes for primary sclerosing cholangitis. Within this series, 55 out of 805 LDLT cases culminated in an iCC diagnosis, with an overall survival rate for LDLT recipients reaching 81.9%. Intriguingly, multivariate analysis identified concurrent cholangiocarcinoma as a significant predictor of mortality (HR: 2.07; 95% CI: 1.71–2.50; p < 0.001) (31). The second largest series was published in 2020 by Bhatti et al., who analyzed the experience in LDLT for cholangiocarcinoma in cases with early stages of the disease and incidental diagnoses. Their study revealed a three-year survival rate of 63% (32). We resume the cases series in **Table 1**, including our first reported case (33–37).

**Table 1:** Summary of the articles evaluating living donor liver transplantation (LDLT) for intrahepatic cholangiocarcinoma (iCC).

Authors	Year	Number	Staged	Setting	Follow-up (months)	Recurrence
**Takatsuki et al.** (33)	2001	1	early	incidental	30	0
**Jonas et al.** (34)	2005	2	advanced	unresectable	46, 35	2
**Sotiropoulos et al.** (35)	2008	1	advanced	unresectable	21	1
Lunsford et al.* (26)	2018	2	advanced	unresectable	36	1
**Hafeez Bhatti et al.** (32)	2020	9	early	incidental	36	not informed
**De Martin et al.** (36)	2020	1	early	incidental	48	not informed
**Rauchfuß et al.** (37)	2020	2	advanced	unresectable	23, 17	0
Sierra et al.** (31)	2023	55	not reported	not reported	not specificated	not informed
**Bednarsch et al.** (38)	2024	1	advanced	unresectable	18	no
Andraus et al***	2024	1	advanced	unresectable	23	no

*Domino’s transplant, **patients with primary sclerosing cholangitis, ***in edition for publication.

A multicentric single-arm clinical trial (NCT04195503) is currently underway to validate LT’s efficacy for stable advanced iCC. This prospective investigation aims to provide conclusive evidence for the therapeutic effectiveness of LT in this context, a crucial step as these findings have not been prospectively verified to date. Of course, one inclusion criterion in this trial is the availability of a compatible living donor (39).

## The first case reported of LDLT for ICC unresectable in Brazil

**Figure 1:**
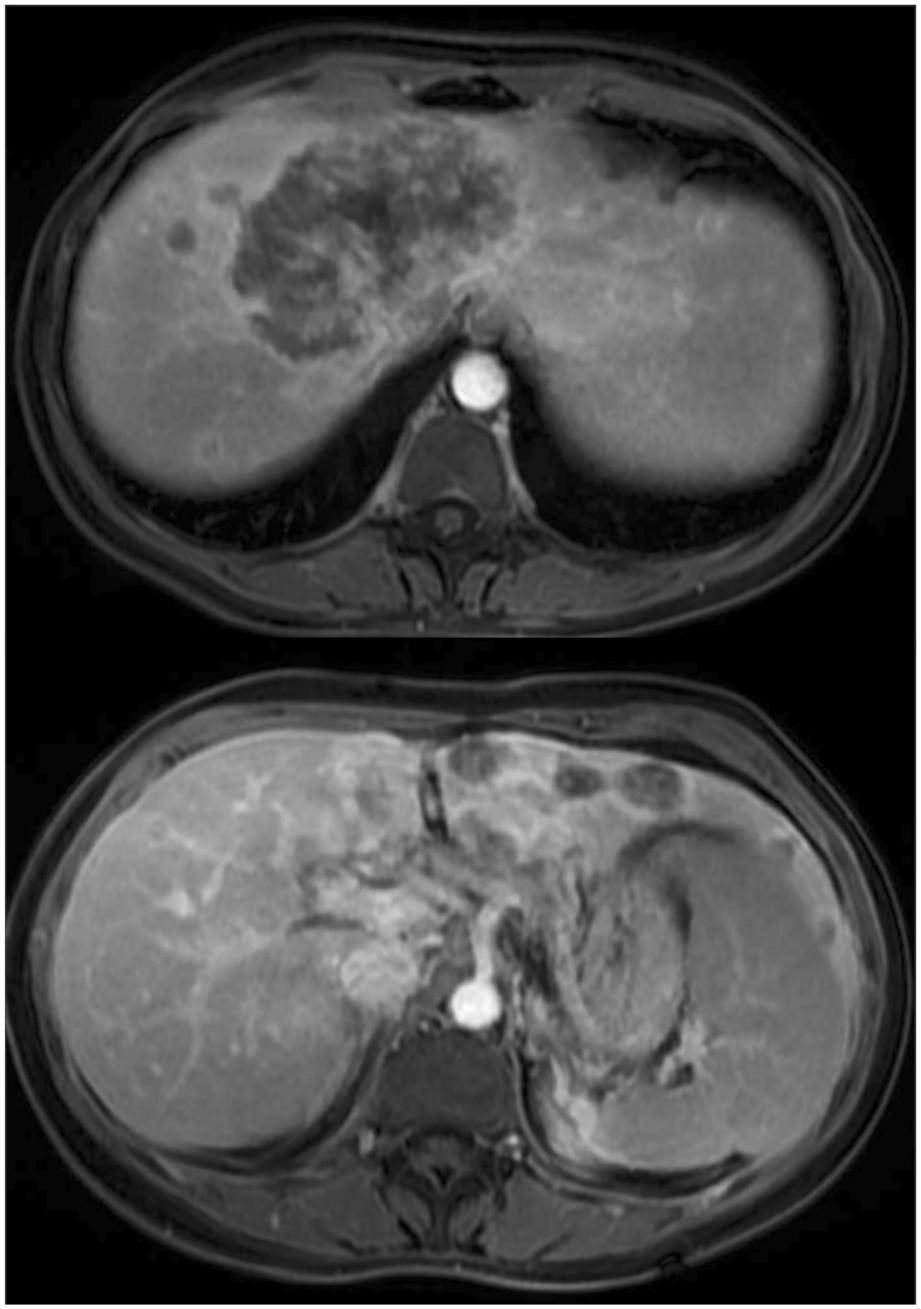
Magnetic resonance imaging showed a multinodular lesion with a predominantly tumor in the central liver with cava vein invasion.

A 36-year-old female patient with no previous medical history presented with upper right abdominal pain. The investigation revealed a multinodular tumor in the liver, characterized by an extensive central tumor affecting the cava vein (**Figure 1**). The CA 19.9 level exceeded 6900 U/mL. A percutaneous biopsy was conducted, confirming the presence of iCC. A multidisciplinary committee deliberated on the unresectable case and opted for systemic therapy using gemcitabine and cisplatin.

There was no significant response after three cycles of systemic chemotherapy. Subsequently, the treatment strategy was altered to intra-arterial chemotherapy with oxaliplatin and gemcitabine, and she underwent a total of eight cycles. CA19.9 exhibited a significant decrease, reaching 216 U/mL, and the tumoral volume was reduced by 20%. Subsequently, she entered a maintenance phase of treatment with intravenous cisplatin and gemcitabine.

Due to the excellent response to intra-arterial treatment, the multidisciplinary board reevaluated the treatment strategy. Despite the positive outcome, the tumor remained unresectable, and LT was considered. The national allocation system in Brazil does not permit the inclusion of iCC in the waiting list. Consequently, the LDLT was an attractive alternative.

A right hepatic lobe LDLT was performed. The donor was his husband. An open surgery was performed. The right hepatectomy was performed, excluding the middle hepatic vein (the vein remained for the donor).

The total liver volume was 1881 cm^3^, and the right lobe volume was 1006 cm^3^. The volume of the remnant liver (left lobe + caudate lobe) was 875 cm^3^ (46.5%). The donor had normal biliary tree anatomy, with one duct after bifurcation to the right lobe. The right hepatic artery originated from the superior mesenteric artery. The donor had a large right hepatic vein, and the middle hepatic vein drained mostly the left lobe.

The graft weight was 922g, and the ratio graft weight/recipient weight was 1.41%. After reperfusion, the graft showed no congested or ischemic areas.

**Figure 2:**
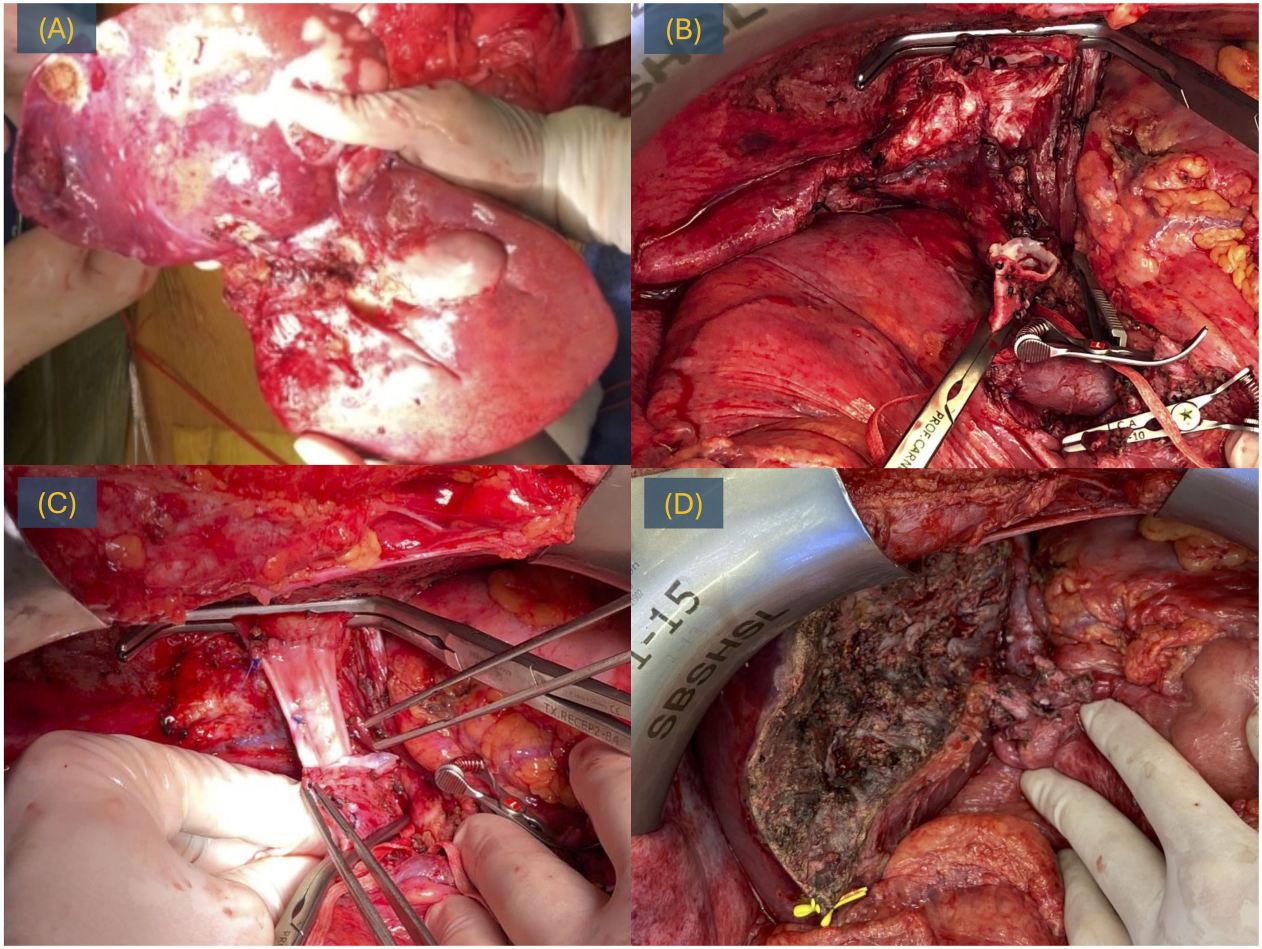
LDLT for iCC, surgery. **(A)** Explant with the tumor. **(B)** Post-hepatectomy time with a cava vein resected. **(C)** Reconstruction of the cava vein with iliac graft. **(D)** Hepatic transection.

Given the complexity of the lesion and the invasion of the cava vein, a veno-venous bypass was executed, and the cava vein underwent resection and reconstruction using an iliac graft (**Figure 2**).

The donor and recipient had an uneventful postoperative course. Pathological analysis of the explant revealed a moderately differentiated, multinodular intrahepatic cholangiocarcinoma, with 30% viable neoplasm and no involvement of the six examined lymph nodes. After 24 months of follow-up, the patient remains alive with no signs of recurrence.

## Discussion

The field of oncologic transplantation has expanded globally, particularly with the extension of indications for primary liver cancer. Initially, the eligibility for transplantation in HCC was confined to the “Milan Criteria,” which selected cases based on size and number (40). Patients meeting the Milan criteria were confirmed as suitable candidates for liver transplantation, leading to an overall survival rate exceeding 70% in five years (41). Currently, it is known that patients who successfully undergo downstage therapy exhibit comparable survival rates to those initially meeting the Milan criteria (42). Other scores incorporating biological indicators and dynamic measures of responsiveness to pre-transplant locoregional therapy and waiting time have been established.

Furthermore, there is mounting evidence indicating that tumor load is just one among numerous variables affecting post-LT outcomes. Recently proposed pre-LT selection criteria have evolved to encompass markers of tumor biology, such as alpha-fetoprotein (AFP) and responsiveness to locoregional treatments. The Working Group Report from the ILTS Transplant Oncology Consensus Conference in 2020 underscored that the selection process should consider tumor biology (including AFP), tumor size, number of tumors, probability of survival, transplant benefit, organ availability, waiting list composition, and allocation priorities (43).

The expansion of LT indications for iCC follows the same steps as HCC. Prior to the 1990s, the concept of biological behavior was unfamiliar, and suboptimal outcomes were attributed to the need for more refined selection criteria. Today, including HCC cases beyond the Milan criteria is widely accepted, particularly in patients exhibiting favorable biological behavior. In the context of iCC, over the last decade, size criteria have been explored, especially for incidental lesions found in LT explants from patients with cirrhosis. It is acknowledged that early-staged lesions of iCC yield comparable oncologic outcomes to early cases of HCC after LT (44). Nevertheless, confining LT indications to early lesions might be overly restrictive. Drawing from the insights gained in the evolution of HCC transplantation, there is a growing perspective that the emphasis should shift toward identifying cases of liver malignancy with favorable biological behavior. This approach could allow for LT as a viable treatment option, irrespective of size and number considerations.

LT in the treatment of iCC is still being defined. Recent publications have proposed highly stringent selection criteria for LT in these patients (18, 19). However, regarding LDLT, the selection process has become even more intricate. Although the risk for the donor must be considered, LDLT offers the advantage of mitigating the impact of an additional indication for LT on the waiting list, presenting itself as a viable option in these non-standard indications.

The 5^th^ edition of the World Health Organization Classification of Tumors, published in 2019, emphasizes the subclassification of iCC based on small and large ducts (45). The small duct type typically manifests in the periphery of the liver and tends to form mass lesions. This subtype shares etiologic and imaging features with HCC, which exhibits better biological behavior (46, 47). Some centers are currently conducting investigations, and certain histological features may contribute to achieving improved outcomes for liver resection or LT in the context of this disease (48, 49).

Certain authors have demonstrated positive outcomes in iCC with LT in patients who responded to neoadjuvant therapy (25, 26). While dropout rates during the waiting period were notable, the tumor’s post-therapy behavior emerged as a crucial parameter for selecting the most suitable candidates for the procedure. In this context, it would be inappropriate to categorically contraindicate LT for all patients with unresectable iCC without considering specific features of the disease, particularly the favorable response observed after neoadjuvant therapy.

The international experience of LT in cases of iCC remains limited to a few centers and is primarily documented in retrospective studies. Only two centers, UCLA and Houston Methodist-MD Anderson, have published prospective findings involving standardized neoadjuvant procedures in patients with preoperatively confirmed iCC (26). Given the current landscape, reaching definitive conclusions about which patients would benefit most from LT for iCC is challenging, but it is clear that the results are promising. Recognizing the significance of international collaboration, contributions to prospective clinical trials encompassing both early and advanced stages of iCC become crucial. Currently, three ongoing trials are actively seeking patients with iCC for LT, two in Canada and one in Norway. These trials recruit individuals with early-staged and unresectable iCC, underscoring the global effort to advance our understanding of LT as a treatment option for this challenging condition (39, 50, 51).

The ideal strategy for expanding LT for iCC would involve using deceased donors (52). Unfortunately, many countries’ low donation rates, particularly Latin America, pose significant challenges. This limited supply of organs fails to meet the demands for traditional indications and hinders the allocation of grafts for new oncological indications like iCC (15, 16). The LDLT is a historical safety strategy for HCC treatment, and now it is being explored for other liver malignancies. Given the rarity of iCC, the selection criteria and the availability of living donors pose additional restrictive factors. Consequently, reports of LDLT for iCC are scarce. Our review identified just eight authors with a short series. Notably, our additional case represents the first report of an LDLT to treat iCC in Latin America. In this instance, we successfully treated a patient with an unresectable advanced tumor compromising the cava vein but with an excellent response to neoadjuvant treatment. In a disease stage where options for potential cure were limited, LDLT with cava resection emerged as the only viable option, and the procedure was highly successful, with no signs of recurrence observed after 24 months of follow-up. This case highlights the effectiveness of selecting candidates based on biological behavior, irrespective of the size and number of tumors.

Currently, there is still a shortage of pieces of evidence in the field of LDLT for iCC. While the favorable outcome of the case we presented is encouraging, it is not sufficient to recommend this therapeutic approach broadly, but rather in carefully selected cases. The challenges related to patient selection, the variability among studies, and the relatively short follow-up period are critical factors that could affect the general applicability of our findings. Despite these limitations, the insights gained from our study provide valuable contributions to the growing body of LDLT for iCC, highlighting its potential as a curative option in selected patients.

## Final comments

There is growing evidence that certain iCC cases may benefit from LT. The key to the success of this approach is a meticulous selection process that identifies patients with the potential for curative treatment. Living donor liver transplantation emerges as a contemporary alternative to broaden the application of LT, particularly in regions facing organ shortages.

## Author contributions

WA: Supervision, Writing – original draft, Writing – review & editing. GO: Data curation, Writing – original draft, Writing – review & editing. RM: Validation, Writing – original draft, Writing – review & editing. RP: Visualization, Writing – original draft, Writing – review & editing. VS: Methodology, Writing – original draft, Writing – review & editing. LL: Investigation, Writing – original draft, Writing – review & editing. RA: Project administration, Writing – original draft, Writing – review & editing. DW: Visualization, Writing – original draft, Writing – review & editing. AS: Formal analysis, Writing – original draft, Writing – review & editing. FT: Conceptualization, Writing – original draft, Writing – review & editing. LC: Supervision, Writing – original draft, Writing – review & editing.
